# Nanocomposite of hydrophobic cellulose aerogel/graphene quantum dot/Pd: synthesis, characterization, and catalytic application†

**DOI:** 10.1039/c9ra01799b

**Published:** 2019-05-31

**Authors:** Sajjad Keshipour, Masoumeh Khezerloo

**Affiliations:** Department of Nanochemistry, Nanotechnology Research Center, Urmia University Urmia Iran s.keshipour@urmia.ac.ir

## Abstract

Novel hydrophobic cellulose aerogel (CA) supported graphene quantum dots (GQD)/Pd were synthesized with high lipophilicity, superior porosity as well as high catalytic activity. The nanocomposite aerogel was obtained in four steps, including transformation of cotton to CA, a silanization reaction of CA in the presence of TiO_2_ nanoparticles to give polysiloxane/TiO_2_ nanoparticles supported on CA (ST@CA), a modification of ST@CA with GQD to yield polysiloxane/TiO_2_ nanoparticles/graphene quantum dots supported on CA (STG@CA), and finally a deposition of Pd nanoparticles on STG@CA. The synthesized aerogel demonstrated hydrophobicity with a water contact angle of 136.2°. It also exhibited excellent oil/water selective absorption capacity with an oil absorption of up to 79 g g^−1^ with 134 g g^−1^ selectivity. Finally, the nanocomposite was used as a heterogeneous catalyst in the oxidation reaction of alcohols, ethylbenzene, and alkenes. High yields, excellent selectivities, green and mild reaction conditions, recyclability and biocompatibility of the catalyst were important features of the reactions.

## Abstract 2

Novel hydrophobic cellulose aerogel (CA) supported graphene quantum dots (GQD)/Pd were synthesized with high lipophilicity, superior porosity as well as high catalytic activity.

## Graphical abstract

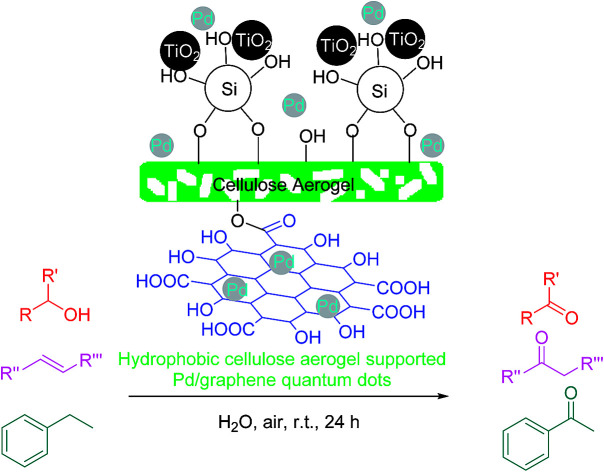

## Introduction

Due to the current lively debate on the importance of the environmental demands of humanity, researchers focused on the use of natural compounds such as cellulose in chemical processes.^1^ A highly porous form of cellulose, known as cellulose aerogel (CA), has attracted a great deal of attention because of application as the catalyst support,^2^ thermal insulating material,^3^ absorbent of pollutants,^4^ and biomedical materials.^5^ CA as the absorbent of organic pollution has shown some advantages such as high absorption capacity (more than 40 times by weight), environmental friendliness, biodegradability, and sustainability.^6,7^ Despite the high potential of CA as an organic substrate absorbent, the polymer has a drawback originating from its inherent hydrophilicity. This contradiction leads to the poor oil/water selectivity of CA. The hydrophilicity of CA can be suppressed by the modification of the polymer surfaces with hydrophobic molecules. Hydrophobization of CA has been reported by chlorosilanes,^8^ triethoxyl(octyl)silane,^9^ methyltrimethoxysilane,^10^ and methyltriethoxysilane.^2*c*^

Graphene quantum dots (GQDs) are zero dimensional graphene composites with lateral dimensions less than 100 nm in single, double or few (3–10) layers.^11^ GQDs have excellent physical and chemical properties such as high surface area, and the best surface grafting *via* the π–π conjugated network. Improved solubility and extended fluorescence are advantages of GQDs compared to those of larger sheets.^6*a*^ GQDs enjoy other advantages such as lower toxicity, resistance to photo bleaching, and biocompatibility compared to the inorganic QDs (for example, CdS and CdTe).^12^ GQDs provide an unprecedented opportunity to improve the performance of catalysts.^6*a*,12,13^ Of particular interest is the recent finding that GQDs can be used as a support for catalysts.^14^ This is because of (a) the low toxicity and biocompatibility of GQDs, (b) increasing the catalyst activity in some of the reactions,^6*a*,12,13^ and (c) easy preparation methods of GQDs such as thermal treatment of citric acid.^14^ Therefore, GQDs are a promising support for heterogeneous catalysts.

Pd is one of the most versatile and ubiquitous catalysts in the oxidation of various organic syntheses. Pd catalyzed oxidation reactions have emerged as the fastest growing field in the organic synthesis because of the high efficiency of this catalyst.^15^ Oxidation of various alcohols,^16^ alkylarenes,^17^ alkanes,^18^ cycloalkanes,^19^ and olefins^20^ were reported with Pd catalysts. A new category of catalysts are hydrophobic materials with extended ability in the organic compounds absorption.^21^ Fluorinated micro–nano hierarchical Pd-decorated on SiO_2_ structure formed by the deposition of Pd nanoparticles (NPs) on SiO_2_ microspheres gave a hydrophobic composite of Pd with water contact angle 170°.^22^ Pd-supported silica nanosphere containing Ti^IV^ and F ions bonded with silicon have been used as a hydrophobic heterogeneous catalyst in one-pot oxidation.^23^ Furthermore, Pd was deposited on a cationic organosilica associated with a hydrophobic anion furnished a hydrophobic catalyst for hydrogenation of alkenes.^24^

The present study was conducted taking into account two important strategies which were recently employed in the designing catalysts including: (a) CA as a biodegradable support of catalysts with excellent absorption capacities, and great surface area provides abundant sites for the catalytic reactions (b) hydrophobic catalysts have insatiable tendency to absorb the organic substrates that elevate the catalyst efficiency. Herein, we silanized CA by methyltrimethoxysilane (MTMS) in the presence of TiO_2_ NPs for increasing the hydrophobicity (or oleophilicity) of the polymer. Then, hydrophobic CA was modified with Pd as a catalyst and GQDs as a co-catalyst.^25^ The CA modified with polysiloxane/TiO_2_ NPs/GQDs/Pd NPs (STGP@CA) is an oleophilic biocompatible heterogeneous catalyst which can be utilized in the oxidation reactions. The catalyst in addition to vast surface area and intense organic absorption ability enjoys from heterogeneity, recyclability, and use of inexpensive sources of compounds for synthesis such as cotton and citric acid.

### Synthesis of STGP@CA

**Scheme 1:**
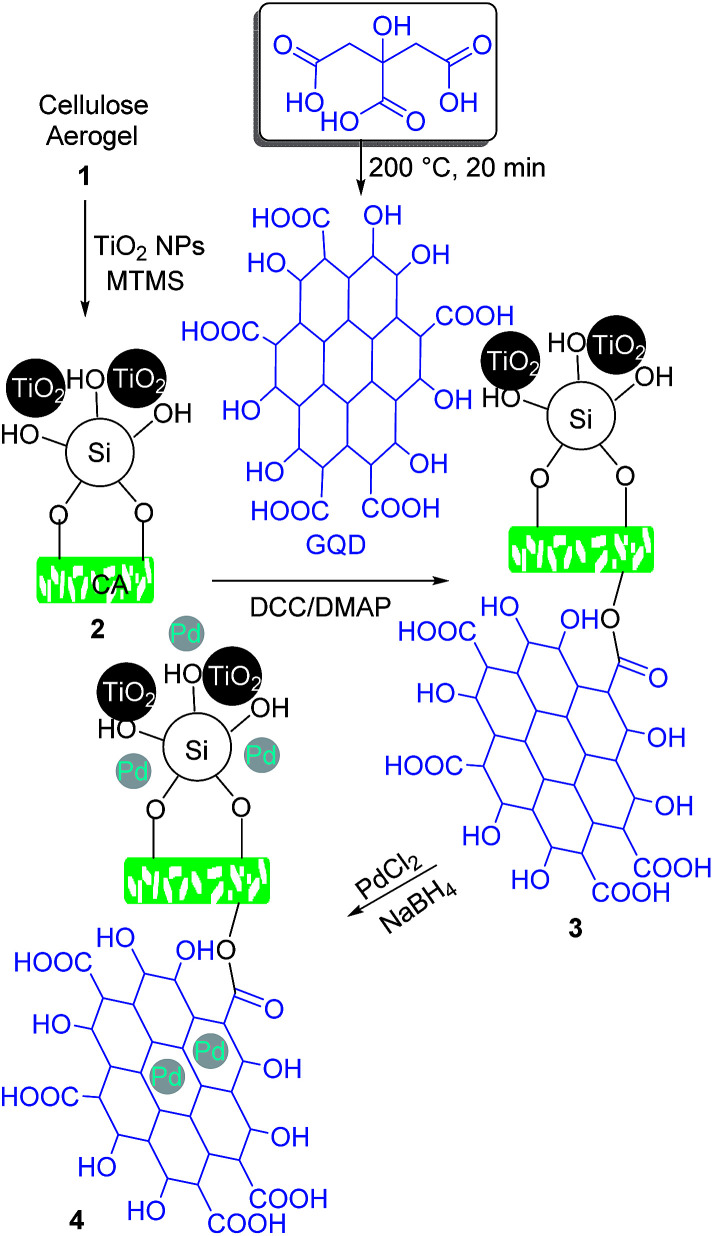
General protocol for the synthesis of STGP@CA.

Since cotton is the abundant and available source of cellulose, it was employed as the precursor of CA according to the procedure explained in the Experimental section. The hydrophobicity of CA was rocketed through the modification of the polymer with MTMS, and TiO_2_ NPs (Scheme 1). The process makes roughness on the polymer giving a sophisticated improve in the hydrophobicity. The coarseness on the polymer acted as an obstacle from direct contact of water drops with the surface. GQDs obtained from the thermal treatment of citric acid were loaded on the CA *via* the esterification reaction promoted with *N*,*N*′-dicyclohexylcarbodiimide (DCC), *N*,*N*′- and dimethylaminopyridine (DMAP).^12*b*^ The new formed bind is chemically stable, which guaranteed retain of GQD on the support during the catalytic reactions of STGP@CA. Finally, *in situ* chemical reduction of Pd(ii) to Pd(0) with NaBH_4_ gave STGP@CA. Deposition of the metal NPs on a support through chemical reduction of metal ions provides a uniform distribution of the catalyst on the support.^26^

### Catalyst characterization

**Fig. 1:**
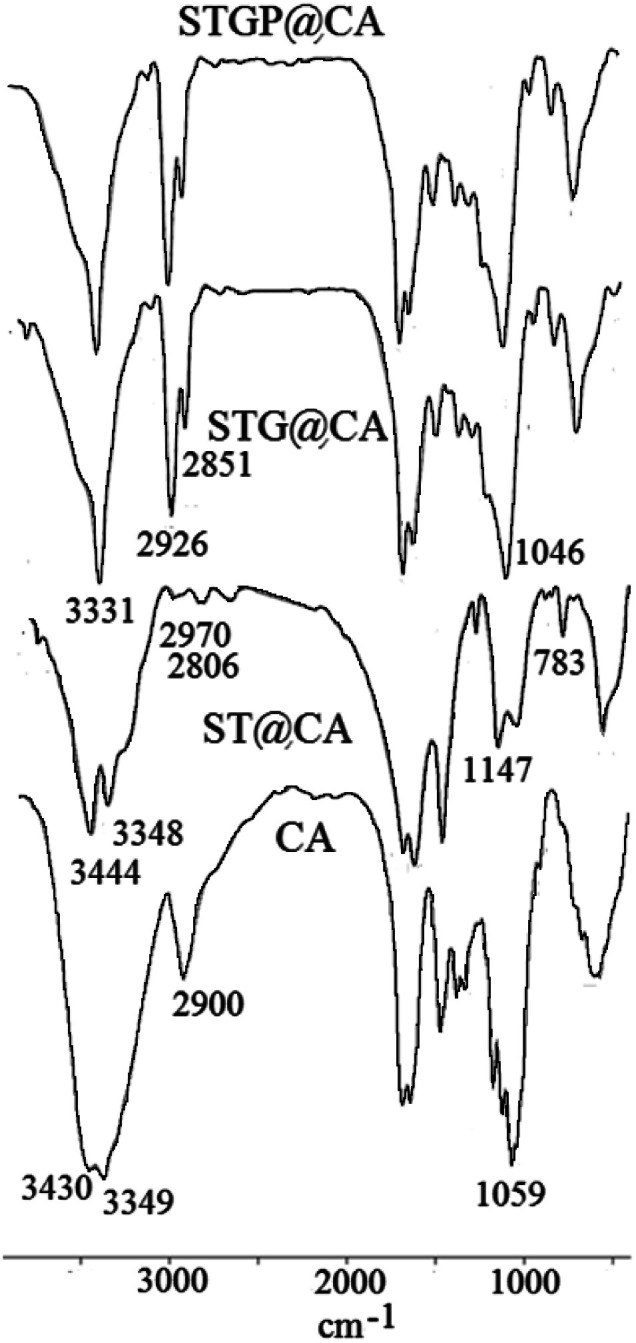
FT-IR spectra of CA, ST@CA, STG@CA, and STGP@CA.

FT-IR spectroscopy is a reliable analysis for confirming the organic transformations in the synthesis of heterogeneous catalysts. FT-IR spectra of CA, ST@CA, STG@CA, and STGP@CA were represented in Fig. 1. The first modification step is the loading of polysiloxane and TiO_2_ NPs on CA which is perceptible through the absorption bands at 1147 cm^−1^ for C–O–Si^27^ and 748 cm^−1^ for Ti–O stretch vibration modes.^28^ In addition, band shifts and change in the peak shapes are observed at above 3300 cm^−1^ arisen from the new hydroxyl groups of polysiloxane and TiO_2_. Absorption bands at 2851 and 2926 cm^−1^ of STG@CA spectrum attributed to the C–H groups of GQDs approves the modification of ST@CA with GQDs (Fig. 1, spectrum STG@CA). Due to the abundance of C–O groups on STG@CA, new absorption band at 1046 cm^−1^ was observed in the related spectrum. Also, the shape of hydroxyl absorption band is changed. The final modification process is the deposition of Pd on STG@CA which did not cause any change on the FT-IR spectrum; therefore this step was confirmed with other approaches.

**Fig. 2:**
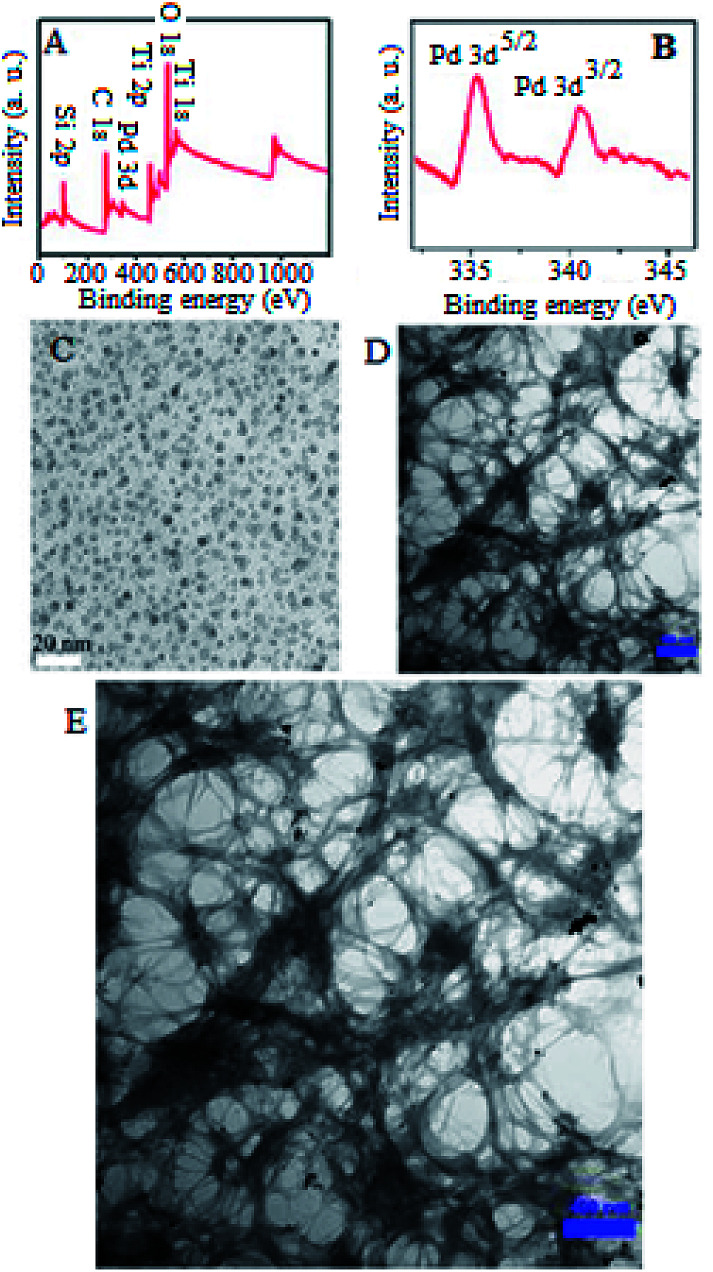
XPS analysis of STGP@CA (A and B) and TEM micrographs of GQD (C), STGP@CA (D), and expansion of (D) (E).

X-ray photoelectron spectroscopy (XPS) analysis was performed to corroborate the complete reduction of Pd(ii) to Pd NPs (Fig. 2). Loading various elements, including Si, Ti, and Pd can be observed in the XPS spectrum. Likewise, the complete reduction of Pd(ii) to Pd(0) can be deduced from the expanded XPS spectra. The expanded XPS spectrum of STGP@CA evinces a Pd 3d^5/2^ doublet and a 3d^3/2^ doublet located at 335.1 and 340.1 eV, respectively. Lack of Pd(ii) peak at 336.5 eV demonstrated the complete reduction of Pd(ii).

TEM micrographs were provided for GQDs and STGP@CA (Fig. 2). A homogeneous synthesis of GQD is approved with TEM micrograph (Fig. 2C). The maximum size distribution of GQDs is between 5.6–7.3 nm. The micrograph of STGP@CA displays the cellulose nanofibers decorated in a porous structure (Fig. 2D). The high porosity caused high surface area which is desirable for the catalytic proposes. Comparison of the nanofibers dimensions for STGP@CA with some cellulose nanomaterials revealed the low diameter of nanofibers in STGP@CA.^10^ Nanoscale dimensions of cellulose fibers on STGP@CA demonstrates the high efficiency of the procedure for providing CA from cotton. In addition, deposition of Pd NPs on STGP@CA can be observed in the transmission electron microscopy (TEM) micrograph with the maximum particles size between 4.1–6.4 nm (Fig. 2E).

**Fig. 3:**
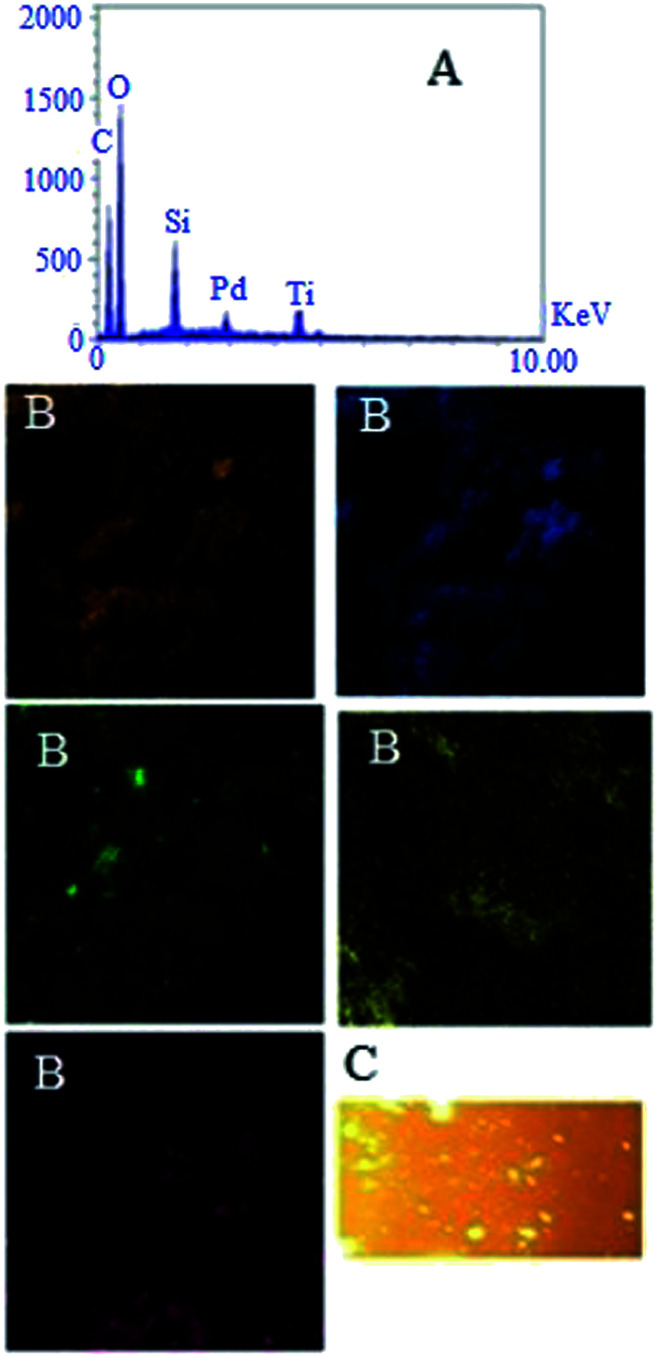
EDX analysis (A), X-ray mapping (B), and fluorescent microscopy of STGP@CA (C).

Energy dispersive X-ray spectroscopy (EDX) analysis showed all the elements introduced in the synthesis of STGP@CA, and also X-ray mapping determined distribution of elements on the support (Fig. 3). Existence of C, O, Si, Ti, and Pd was corroborated with EDX. Investigation of the mapping from catalyst surfaces evinced homogeneous distribution of C (orange), O (blue), Si (green), Ti (yellow), and Pd (purple) on the support. The homogeneous distribution of Si, and Ti on CA confirmed the success of uniform hydrophobization process on the catalyst surfaces. Furthermore, monotonic Pd distribution on the composite afforded a heterogeneous catalyst with highly available surfaces. Thus, the preparation of hydrophobic heterogeneous catalyst was performed successfully with the Pd loading 0.036 g (0.18 mmol) per 1 g of the catalyst pursuant to flame atomic absorption spectroscopy (FAAS).

The grafting GQD on STGP@CA can be surveyed by fluorescent properties of the synthesized nanocomposite because of GQD high fluorescent activity. The fluorescence microscopy of STGP@CA showed high fluorescence which approves the loading GQD on STGP@CA (Fig. 3).

In addition, comparison of the CHN elemental analyses of ST@CA with STG@CA demonstrates differences which attributed to the GQD deposition on STG@CA. According CHN analyses, STG@CA with 37.46% C and 5.62% H have higher content of carbon compared to ST@CA with 37.11% C and 5.74% H which approves the GQD loading on STG@CA.

**Fig. 4:**
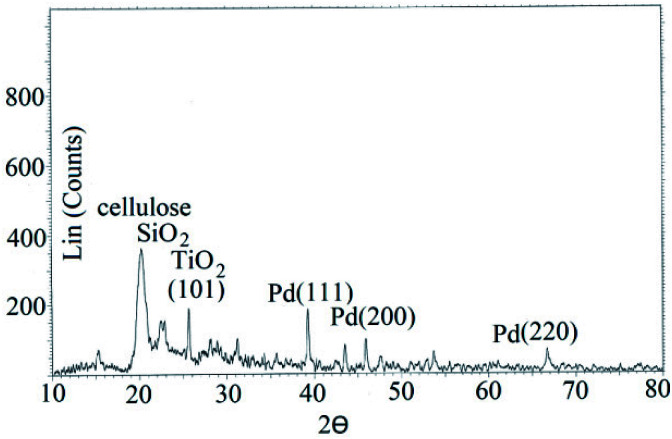
XRD pattern of STGP@CA.

XRD pattern of STGP@CA showed characteristic peaks of cellulose, and SiO_2_. Pd nanocrystals indicated diffraction peaks related to plans (111), (200), and (220). Finally, TiO_2_ NPs have diffraction peak of (101) (Fig. 4).

### Wettability

The wettability of STGP@CA was characterized by the WCA of the aerogel surface. Unlike CA, all of its modified forms, including ST@CA, STG@CA and STGP@CA exhibited hydrophobic properties. While CA is hydrophilic with water absorption capacity of 49 g, the ST@CA is a highly hydrophobic nanostructure with WCA above 152.0°. WCA is ruled by the surface energy and the surface roughness. The surface energy of the CA was promoted by the silanization reaction. Likewise, the formation of polysiloxane particles and deposition of TiO_2_ NPs make high surface roughness of ST@CA. The STG@CA displayed a WCA of up to 140.1°, which was lower than WCA of ST@CA. Therefore, the GQDs entrance into nanocomposite declined the WCA which may be ascribed to the hydrophilic behaviour of GQDs. Moreover, the WCA of STG@CA was decreased with the deposition of Pd NPs, exhibiting a WCA of up to 136.2°. The oleophilicity of STG@CA was evaluated by dripping oil drops on the surface of the STGP@CA which indicated lipophilicity (ESI, Video 1†).

**Fig. 5:**
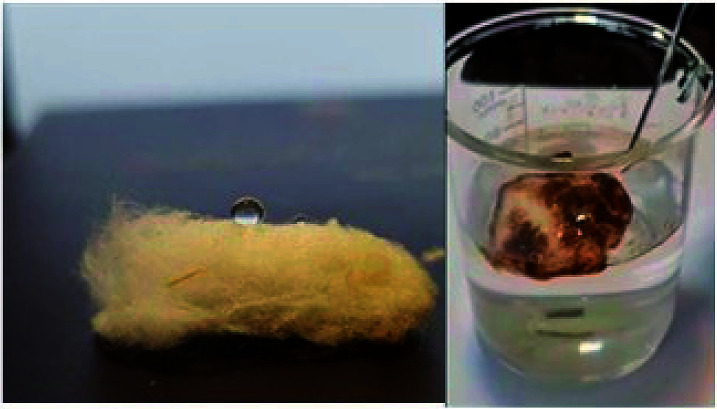
Water and oil contact with STGP@CA.

Owing to its hydrophobicity and lipophilicity, STGP@CA is potential candidates for the separation of oils and organic pollutants from water. When the STGP@CA was placed in oil/water mixture solution, it selectively absorbed the oil, leaving the clean water (Fig. 5). The STGP@CA can be float on the water surface after absorbing the oil due to its low density. Also, the capacity of STGP@CA was surveyed for the oil which 79 g g^−1^ with 134 g g^−1^ selectivity of oil per water was obtained. The capacity and selectivity were examined for 1-octanol, ethylbenzene, styrene, 1-hexene, and cyclohexene (Table 1). STGP@CA efficiently absorbed all of the substrates with some low efficiency for 1-octanol which may be attributed to partially hydrophilic property of the alcohol. In addition, absorptions of hydrophilic alcohols such as 2-propanol, cyclohexanol, and 1-pentanol by STGP@CA from water were unsuccessful due to their affinity to water.

**Table 1:** Capacity and selectivity of STGP@CA in the separation of organic substrates

Entry	Organic substrate	Capacity (g)	Selectivity (g g^−1^ of water)
1	1-Octanol	57	68
2	Ethylbenzene	69	106
3	Styrene	68	99
4	1-Hexene	71	122
5	Cyclohexene	73	129

### Oxidation of alcohols, ethylbenzene, and alkenes

The catalytic activity of STGP@CA was investigated in the oxidation reactions of alcohols, ethylbenzene, and alkenes under air as the oxidant (bubbling rate 20 ml min^−1^). The oxidation reaction conditions were optimized through changing the catalyst amounts and the reaction media for the oxidation of 1-pentanol (1 ml). At the end of reaction, gas chromatography (GC) analysis of the solution showed pentanoic acid as the main product. The selective production of pentanoic acid is interesting, since the reaction can be lead to the formation of pentanal. The conversion and selectivity of this reaction were determined by GC analysis 84% and 98.1%, respectively. Turnover number (TON) was calculated 214 for the oxidation reaction of 1-pentanol. The reaction gave highest yield in the presence of 0.036 mmol of the catalyst and any further yield did not obtain in the high catalyst loading. The solvent effect was investigated for the aerobic oxidation reaction of 1-pentanol. Among the various screened solvents, H_2_O is the best solvent regarding both high yield and non-toxic inherent (Table 2).

**Table 2:** Solvent effect on the oxidation of benzyl alcohol^a^

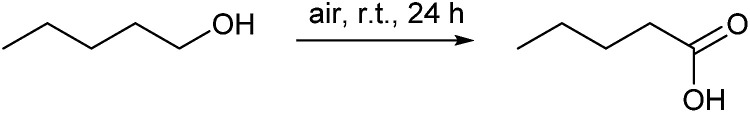			
Entry	Catalyst (mmol)	Solvent	Conversion (%)
1	0.018	H_2_O	69
2	0.036	H_2_O	84
3	0.054	H_2_O	84
4	0.036	—	77
5	0.036	EtOH	51
6	0.036	MeOH	59
7	0.036	CH_3_CN	63
8	0.036	PhCH_3_	72
9	0.036	CH_2_Cl_2_	18

a Reaction conditions: 1-pentanol (1 ml), catalyst, solvent (10 ml), air (bubbling rate 20 ml min^−1^), r.t., 24 h.

Examination of the catalyst activity for other aliphatic alcohols such as 1-octanol, 2-propanol, and 2-butanol lead to the formation of corresponding carboxylic acids. While the oxidation reaction of 1-octanol gave high selectivity, TON about 138 for this reaction indicates hard oxidation reaction of the large molecules. For some of the substrates such as 2-propanol and 2-butanol a product is conceivable, and the corresponding ketones were achieved. TONs for these reactions are higher than 1-pentanol and 1-octanol which evince easier oxidation of secondary alcohols compared to primary alcohols. Oxidation of benzyl alcohol (2 ml) gave benzoic acid selectively with 314 TON. High TON of the reaction is related to the easy oxidation of aromatic alcohols (Table 3).

**Table 3:** Oxidation of various substrates with STGP@CA^a^

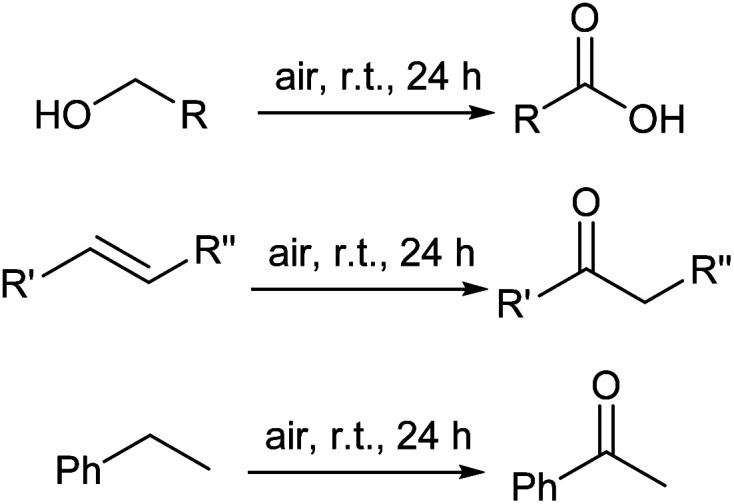				
Entry	Substrate	Conversion (%)	Selectivity (%)	TON
1	1-Pentanol	84	98.1	214
2	1-Octanol	79	97.9	138
3	2-Propanol	83	100	300
4	2-Butanol	88	100	266
5^b^	Benzyl alcohol	59	98.8	314
6	Ethylbenzene	67	100	150
7^b^	Styrene	63	97.5	304
8	1-Hexene	71	93.6	157
9	Cyclohexene	68	94.8	185

a Reaction conditions: substrate (1 ml), catalyst (0.036 mmol), H_2_O (10 ml), air (bubbling rate 20 ml min^−1^), r.t., 24 h.

b Substrate (2 ml).

Ethylbenzene (1 ml) was oxidized in similar conditions to afford acetophenone in excellent selectivity with 150 TON (Table 3, entry 6). The low yield of reaction is related to the difficult oxidation of alkylarenes compared to alcohols.

Styrene (2 ml), 1-hexene (1 ml), and cyclohexene (1 ml) oxidation in same reaction conditions with alcohols gave the corresponding ketones (Table 3, entries 7–9). The results exhibit styrene can be oxidized by STGP@CA catalyst with quantitative conversion due to the easy oxidation of aromatic substituted alkenes. The oxidation of 1-hexene and cyclohexene afforded moderate conversions which show difficult oxidation of linear- and cyclo-alkenes.

For discovery of the catalytic effects of GQDs and Pd deposited on the STGP@CA, oxidation reactions of benzyl alcohol, ethylbenzene, and styrene were carried out with STG@CA (the catalyst without Pd) and STP@CA (the catalyst without GQD). The results are summarized in Table 4. The oxidation reactions did not perform in the absence of Pd (Table 4, entries 1–3). In addition, low catalytic activity of Pd for the oxidation reactions was observed in the absence of GQD. Therefore, GQD enhances the catalyst activity of Pd NPs for the oxidation reactions.^23^ Furthermore, the support effect on the reaction was studied *via* performing the oxidation of benzyl alcohol with cellulose supported catalyst (STGP@C) instead of CA which low yields obtained. This result is related to the low surface area of cellulose that provided limited sites for catalyst compared to CA support. Besides, the reaction was examined using catalytic amounts of GP@CA for distinguishing the lipophilicity effect of the support on the reaction yield. Interestingly, the reaction yield was declined using GP@CA. Therefore, the lipophilicity of the nanocomposite augments the reaction yield due to great tendency of the nanocomposite to absorption of the organic substrates. These results support a new concept in the catalytic reactions including the effectiveness of lipophilicity of catalysts on the catalytic transformations for the organic substrates.^23,24^

**Table 4:** Oxidation of benzyl alcohol, ethylbenzene, and styrene with STGP@CA, STG@CA, STP@CA, STGP@C, and GP@CA^a^

Entry	Substrate	Catalyst	Conversion (%)
1	Benzyl alcohol	STGP@CA	59
2	Ethylbenzene	STGP@CA	67
3	Styrene	STGP@CA	63
4	Benzyl alcohol	STG@CA	0
5^b^	Ethylbenzene	STG@CA	0
6	Styrene	STG@CA	0
7	Benzyl alcohol	STP@CA	47
8^b^	Ethylbenzene	STP@CA	42
9	Styrene	STP@CA	51
10	Benzyl alcohol	STGP@C	54
11	Benzyl alcohol	GP@CA	52

a Reaction conditions: substrate (2 ml), catalyst (0.036 mmol), H_2_O (10 ml), air (bubbling rate 20 ml min^−1^), r.t., 24 h.

b Substrate (1 ml).

Potential Pd leaching into the mixture of benzyl alcohol oxidation reaction was also analyzed with FAAS analysis. For this purpose, the filtrate was taken through a syringe filter during the heterogeneous oxidation reaction of benzyl alcohol after 10 min was dissolved in HNO_3_. The FAAS analysis of sample showed the Pd concentration in the reaction mixture was less than the detection limit. This result indicates that virtually no Pd leaches from STGP@CA into the mixture.

### Recyclability of STGP@CA

The recyclability of STGP@CA was evaluated in the oxidation reaction of benzyl alcohol (2 ml). After carrying out the reaction, the catalyst was separated by filtration as a white solid, washed with acetone (2 × 5 ml), dried in oven at 70 °C, and reused. A minor decrease in the reaction yield was observed after six cycles for the catalyst which indicates recyclability of STGP@CA in the oxidation reaction (Table 5). In addition, the catalyst showed high stability in the reaction mixture and a negligible diminution in the recovered catalyst amount was observed. For the investigation of Pd stability on the support, the amount of Pd on the catalyst was specified before use and after six runs which 0.036 mmol per 1 g catalyst achieved for both of them.

**Table 5:** Recyclability of STGP@CA in the oxidation of benzyl alcohol^a^

Entry	Recovered catalyst (mg)	Conversion (%)
1	0.19	59
2	0.19	59
3	0.19	59
4	0.19	59
5	0.18	57
6	0.18	53

a Reaction conditions: benzyl alcohol (2 ml), catalyst (0.036 mmol), H_2_O (10 ml), air (bubbling rate 20 ml min^−1^), r.t., 24 h.

### Comparison of the results

A comparison of the acquired results with the previous reports will elucidate the importance of STGP@CA. To the best of our knowledge, there isn't any report about the lipophilic catalysts for the oxidation of alcohols, ethylbenzene, and alkenes. Therefore, the comparisons were carried out separately for oil absorption capacity and oxidation catalytic activity of STGP@CA. Table 6 describes some recent synthesized materials for the oil absorption. Modification of banana cellulose with succinic anhydride furnished an oleophilic compound with 32 g g^−1^ capacity; the low capacity of this absorbent attributed to its non-porous structure. Composite of CA with polyvinyl alcohol afforded a potent absorbent for oil. Magnetic/silanized cellulose sponges have a little structurally differences with STGP@CA related to employing magnetic nanoparticles instead of titania. Surprisingly, STGP@CA showed high oil absorption capacity compared to magnetic/silanized cellulose sponges which attributed to great hydrophobicity of titania NPs *versus* iron oxides.

**Table 6:** Comparison of the oil absorption capacity

Entry	Absorbent	Oil absorption capacity (g g^−1^)
1	STGP@CA	79
2	Succinic anhydride modified banana cellulose	32
3	Polyvinyl alcohol/cellulose nanofibril hybrid aerogel microspheres	116
4	Magnetic/silanized ethyl cellulose sponges	37–51

Comparison of the results for the benzyl alcohol oxidation in the presence of various catalysts revealed high catalytic activity of STGP@CA (Table 7). While each of the reactions in Table 7 have some advantages, oxidation reaction with STGP@CA catalyst is interesting because of supreme yield, green oxidant, non-toxic solvent, and mild reaction conditions.

**Table 7:** Comparison of the reaction conditions and yield for the various catalytic oxidation of benzyl alcohol

Entry	Catalyst	Solvent	Temp. (°C)	[ox]	Time (h)	Yield (%)
1	STGP@CA	H_2_O	r.t.	Air	24	97
2	Nano Pd(0)@cellulose	CH_3_CN	80	Air	15	82
3	RuNPs/CNFs	Toluene	110	Air	24	89
4	Au(iii)/dimercaprol@chitosan	Solvent-free	r.t.	H_2_O_2_	1	96

## Conclusion

In conclusion, an aerogel of cellulose was synthesized and modified with polysiloxane/titania, graphene quantum dot, and Pd NPs to give a hydrophobic compound with an oil absorption ability in high efficiency. The nanocomposite was utilized in the catalytic oxidation of alcohols, ethylbenzene, and alkenes to afford the corresponding ketones or carboxylic acids. The catalyst is recyclable, and no catalyst leaches into the reaction mixture, which demonstrates the chemical stability of the new catalyst. As the most important result of the research, hydrophobization of the heterogeneous catalyst led to the high efficiency of the catalyst in the oxidation reactions. This matter can be expanded to the other catalytic systems.

## Experimental

### Materials and methods

All reagents were purchased from Sigma-Aldrich and used without further purification. Cotton was purchased from local market as a Turkish cotton. Citric acid and TiO_2_ NPs with particle size < 100 nm were provided from Sigma-Aldrich Company. ScanVac CoolSafe 95–15 Pro freeze-dryer (Denmark) carried out freeze-drying process. XPS and EDX were performed by VG multilab 2000 spectrometer (ThermoVG scientific) and Scanning Electron Microscope of TSCAN Company, respectively. TEM micrograph was obtained with Philips CM100 BioTWIN transmission electron microscope. Pd determination was carried out on a FAAS (Shimadzu 105 model AA-680 atomic absorption spectrometer) with a Pd hollow cathode lamp. The elemental analysis was performed with an Elementar Analysensysteme GmbH VarioEL. Fluorescence microscope KERN OBN-14 gave fluorescence image. Gas chromatographic (GC) measurements were carried out in Varian 3900 GC. WCA measurement was performed by OCA20. The following conditions were used for all GC analyses: injector and detector temperature, 260 °C; initial temperature, 100 °C; temperature ramp, 3 °C min^−1^; final temperature, 280 °C.

### Preparation of STGP@CA

For the preparation of CA, cotton (2 wt%) was dispersed into a 50 ml sodium hydroxide/urea solution (1.9 wt%/10 wt%) by sonication for 6 min. Then, the solution was placed in a refrigerator at 4 °C for about 24 h to allow gelation. Then, it was thawed at room temperature for 5 h, followed by immersion into ethanol (99 vol%) for coagulation. Immersing the gel in deionized water for 2 days gives the opportunity to the solvent exchange process. Freeze-drying was carried out for the sample during 2 days at −48 °C to obtain CA (1.4 g).

For the modification of CA with polysiloxane and titania NPs (ST@CA), a mixture of CA (2.0 g), TiO_2_ NPs (0.05 g), and MTMS (5 ml) was heated to 60 °C, aged for 2 h, and washed with acetone (3 × 5 ml). The obtained white solid (2.12 g) was dried in vacuum at 60 °C for 5 h.

The chemical modification of ST@CA with GQDs gave STG@CA. A heater-stirrer mixed a balloon containing ST@CA (2.0 g), DCC (0.5 g), DMAP (0.1 g), and GQDs (0.5 g) in DMSO (10 ml) at 90 °C for 24 h. GQD was prepared from heating citric acid at 200 °C during 15 min.^14^ Finally, the precipitate of mixture was filtered off, washed with acetone (3 × 5 ml), and dried in vacuum at 60 °C for 5 h to give white solid (2.1 g).

For the preparation of STGP@CA, a mixture of STG@CA (2.0 g) and PdCl_2_ (0.05 g) in H_2_O (20 ml) was stirred at room temperature for 24 h. Then, addition of 10 ml solution of NaBH_4_ (0.1 g) during 0.5 h to the reaction vessel and stirring the mixture for 3.5 h gave STGP@CA (2.0 g) after filtration, washing with water (20 ml), and drying the product in the vacuum oven at 70 °C as a cream color solid (2.0 g).

### Determination of Pd on STGP@CA using FAAS

A mixture of STGP@CA (0.05 g) and HCl : HNO_3_ (3 : 1) (10 ml) was sonicated for 3 h. Then, the mixture was filtered off and the total volume of the filtrate made up to about 20 ml with distilled water. The final solution was aspirated into the flame of the AAS against the blank prepared with STG@CA. The Pd concentration was obtained using calibration curve prepared with Pd solution standards.

### Wettability and liquid absorption capacity of STGP@CA

WCA measurement (OCA20) equipped with a high-speed camera indicated the surface wettability of STGP@CA. Water droplets (1 μl) were deposited on the surface of the aerogels and the static contact angles measured immediately. For liquid absorption test, STGP@CA (0.1 g) was immersed into 50 ml oil for a certain time and then picked out for the measurements. The weight of STGP@CA filled with liquids after the aerogel wiped with a filter paper to remove excess liquids can be used in the eqn (1) for determining of the liquid absorption capacity:

1 *C* = (*W*_1_ – *W*_0_)/*W*_0_

where *W*_0_ and *W*_1_ are the weights of STGP@CA before and after absorption, respectively.

### Typical procedure for the oxidation of ethylbenzene

A stirrer mixed ethylbenzene (1 ml), STGP@CA (0.036 mmol or 0.2 g), and H_2_O (10 ml) at room temperature under air bubbling rate 20 ml min^−1^ for 24 h. The progress of the reaction was monitored by thin layer chromatography (TLC). Upon completion, the catalyst was separated *via* filtration and the mixture analyzed with GC.

## Conflicts of interest

There are no conflicts to declare.

## Supplementary Material

RA-009-C9RA01799B-s001

RA-009-C9RA01799B-s002
