# Supplementary material for: Nanocomposite of hydrophobic cellulose aerogel/graphene quantum dot/Pd: synthesis, characterization, and catalytic application
Source: RSC Adv. 2019 May 31;9(30):17129–36. doi: 10.1039/c9ra01799b (PMC9064462; doi:10.1039/c9ra01799b)
Supplement: RA-009-C9RA01799B-s001 [file RA-009-C9RA01799B-s001.pdf]

Electronic Supplementary Material (ESI) for RSC Advances.
This journal is © The Royal Society of Chemistry 2019

# Supporting Information:

# Nanocomposite of hydrophobic cellulose aerogel/graphene quantum dot/Pd: Synthesis, characterization, and catalytic application

Sajjad Keshipour,* and Masoumeh Khezerloo

*Department of Nanochemistry, Nanotechnology Research Center, Urmia University, Urmia, Iran;*

E-mail: s.keshipour@urmia.ac.ir

**1H NMR spectra:**

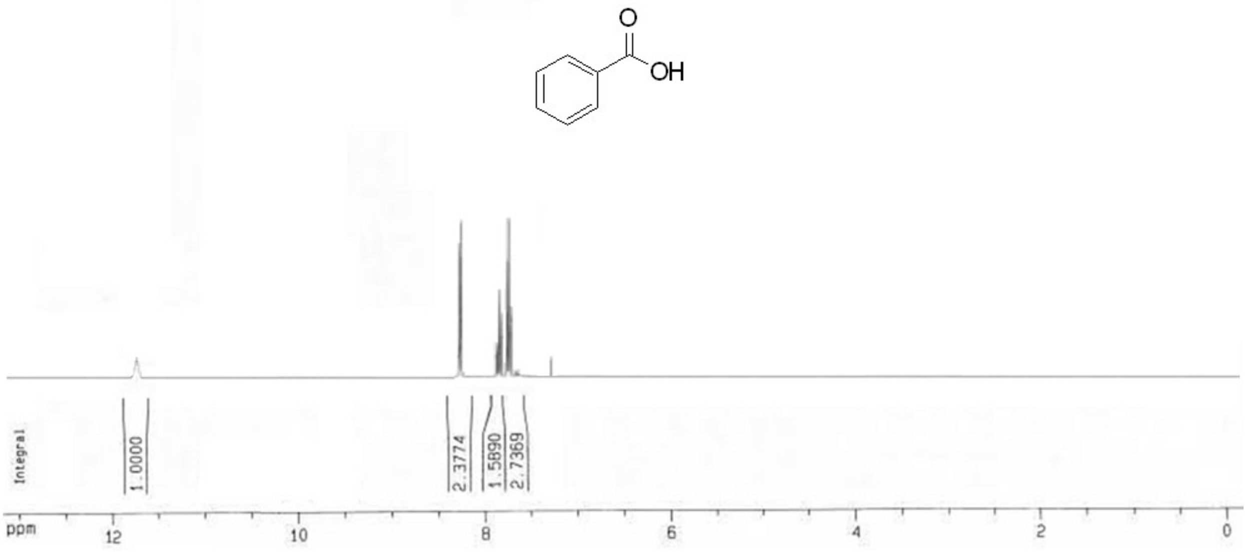


**Fig. 1** Product of benzyl alcohol oxidation

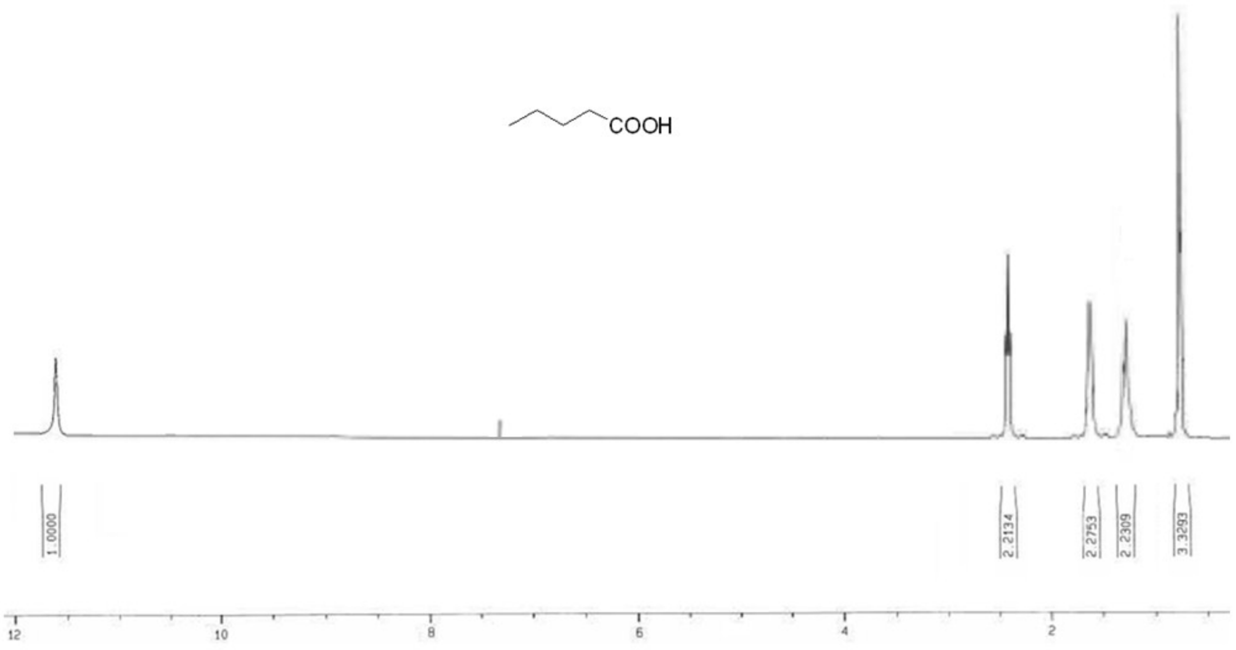


**Fig. 2** Product of 1-pentanol oxidation

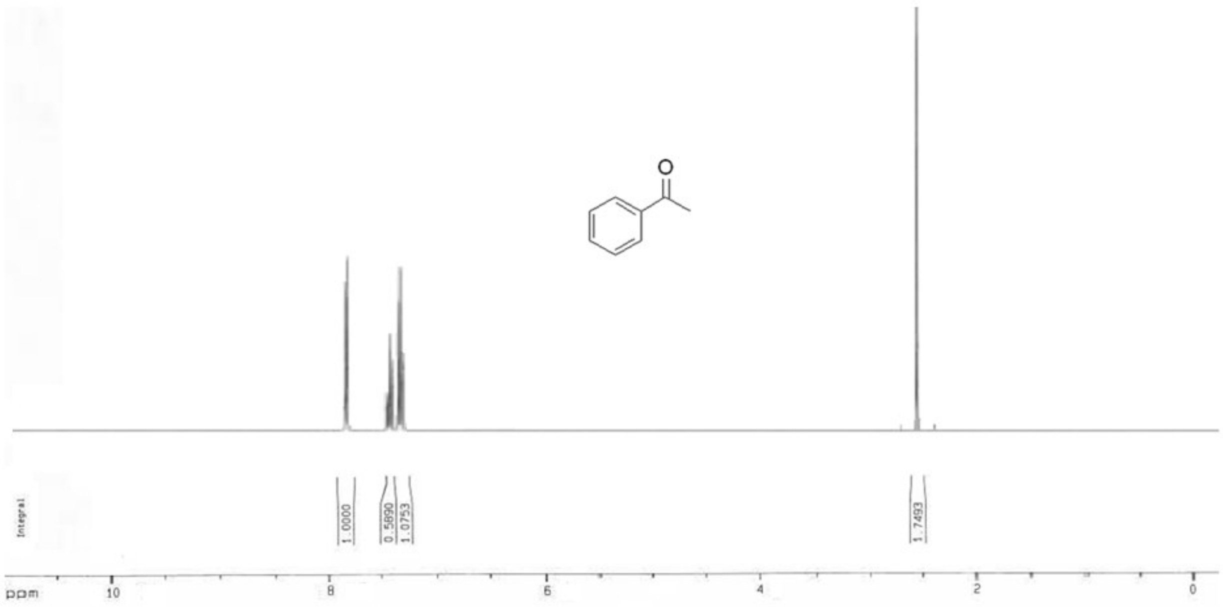


**Fig. 3** Product of ethylbenzene oxidation

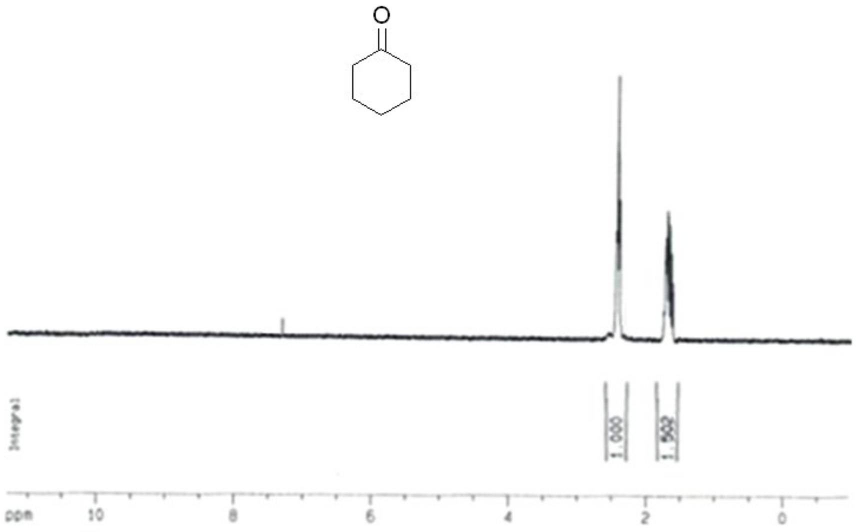


**Fig. 4** Product of cyclohexene oxidation
